# Anticancer and Anti-Inflammatory Activities of a Standardized Dichloromethane Extract from *Piper umbellatum* L. Leaves

**DOI:** 10.1155/2015/948737

**Published:** 2015-02-03

**Authors:** Leilane Hespporte Iwamoto, Débora Barbosa Vendramini-Costa, Paula Araújo Monteiro, Ana Lúcia Tasca Gois Ruiz, Ilza Maria de Oliveira Sousa, Mary Ann Foglio, João Ernesto de Carvalho, Rodney Alexandre Ferreira Rodrigues

**Affiliations:** ^1^Department of Pharmacology, Anaesthesiology and Therapeutics, Faculty of Dentistry, University of Campinas, Avenida Limeira 901, 13414-903 Piracicaba, SP, Brazil; ^2^Chemical, Biological and Agricultural Pluridisciplinary Research Center (CPQBA), University of Campinas, Rua Alexandre Cazelatto 999, Vila Betel, 13148-218 Paulínia, SP, Brazil; ^3^Department of Organic Chemistry, Institute of Chemistry, University of Campinas, Rua Josué de Castro s/n, Cidade Universitária Zeferino Vaz, Barão Geraldo, 13081-970 Campinas, SP, Brazil; ^4^Faculty of Pharmaceutical Sciences, University of Campinas, Cidade Universitária Zeferino Vaz, 13081-970 Campinas, SP, Brazil

## Abstract

Despite the advances in anticancer drug discovery field, the worldwide cancer incidence is remarkable, highlighting the need for new therapies focusing on both cancer cell and its microenvironment. The tumor microenvironment offers multiple targets for cancer therapy, including inflammation. Nowadays, almost 75% of the anticancer agents used in chemotherapy are derived from natural products, and plants are an important source of new promising therapies. Continuing our research on *Piper umbellatum* species, here we describe the anticancer (*in vitro* antiproliferative activity and *in vivo* Ehrlich solid tumor model) and anti-inflammatory (carrageenan-induced paw edema and peritonitis models) activities of a standardized dichloromethane extract (SDE) from *P. umbellatum* leaves, containing 23.9% of 4-nerolidylcatechol. SDE showed *in vitro* and *in vivo* antiproliferative activity, reducing Ehrlich solid tumor growth by 38.7 and 52.2% when doses of 200 and 400 mg/kg, respectively, were administered daily by oral route. Daily treatments did not produce signals of toxicity. SDE also reduced paw edema and leukocyte migration on carrageenan-induced inflammation models, suggesting that the anticancer activity of SDE from *Piper umbellatum* leaves could involve antiproliferative and anti-inflammatory effects. These findings highlight *P. umbellatum* as a source of compounds against cancer and inflammation.

## 1. Introduction

Nature has been a source of medicinal products for millennia, going along with the history of humanity. Due to the improvement on methods for isolation, identification, and synthesis during the last century, many drugs have arisen from natural sources. In chemotherapy field, around 75% of the anticancer agents used nowadays are derived from natural products of different origins, including plants, microorganisms, and marine organisms [1]. One important example of natural source is the* Piper* genus (Piperaceae family), which comprises approximately 2000 species, distributed mainly in tropical areas and widely evaluated for their medicinal properties [2].* Piper umbellatum* L. (syn.* Pothomorphe umbellata* (L.) Miq.,* Lepianthes umbellata* (L.) Raf.,* Heckeria umbellata* (L.) Kunth, and* Peperomia umbellata* (L.) Kunth) is a perennial shrub or woody herb, popularly known in Brazil as pariparoba, caapeba, and malvarisco [3]. Other synonyms for* Piper umbellatum* L. have been suggested, although some of them are still under revision (available on http://www.theplantlist.org/tpl1.1/record/kew-2571246).

This species was included in the Brazilian Pharmacopoeia first edition (1926) and 94 traditional medicinal uses for* P. umbellatum* are registered [3]. Indeed, there are several pharmacological activities described, such as antioxidant [4], anti-inflammatory and analgesic [5], antibacterial [6], antifungal [7], antitumor [8] activities and protection against photoaging [9] and anticonvulsants [10]. Moreover, phytochemical studies of* P. umbellatum* leaves extracts have demonstrated the presence of terpenes, alkaloids, flavonoids, and sterols, the catechol 4-nerolidylcatechol (4-NC) being the majority compound [3, 6, 11].

Previous studies performed by our group evaluated the* in vitro* and* in vivo* anticancer activities of a dichloromethane crude extract (DCE) from* P. umbellatum* leaves and its fractions, showing that intraperitoneal (i.p.) treatment with DCE (200 mg/kg) increased the life span of Ehrlich ascitic tumor-bearing animals and that treatment with a higher dose (400 mg/kg) promoted toxicity [8]. Another study conducted by our group demonstrated that* Piper regnellii* DCE and its fractions inhibited Ehrlich solid tumor development in mice [12].

The emergence of a cancer (carcinogenesis) is a complex and multistep process during which normal cells progressively acquire a neoplastic phenotype. Each genetic modification confers to tumor cells a type of advantage, constituting the hallmarks of cancer, such as self-sustained proliferation, evasion of growth signals suppressors, resistance to cell death, limitless replication, inducing angiogenesis, and activating invasion and metastasis processes [13]. Besides cancer hallmarks, the tumor microenvironment also influences cancer development, and one prominent microenvironment stimulus in carcinogenesis is inflammation [14].

Despite the advances in the field of anticancer drug discovery, the statistics are noteworthy; in 2012, 14.1 million new cases of cancer were diagnosed worldwide, with 8.2 million deaths [15]. Thus, there is still a necessity for the development of new therapies and the tumor microenvironment is an important source of multiple targets for cancer therapy, including inflammation [16].

Bearing in mind the need for new therapies, specially focusing on the tumor microenvironment and the potential of* Piper umbellatum* as an anticancer agent, in this study we evaluated the* in vitro* and* in vivo* antiproliferative activities of a standardized dichloromethane crude extract (SDE) from* P. umbellatum* leaves, containing 23.9% of 4-NC. We also evaluated its anti-inflammatory activity, looking for evidences of the relationship between the SDE anticancer and anti-inflammatory activities.

## 2. Materials and Methods

### 2.1. Plant Material

*Piper umbellatum* leaves were collected in February 2013 at an experimental field of the Chemical, Biological and Agricultural Pluridisciplinary Research Center (CPQBA, UNICAMP, Paulínia, SP, Brazil). A voucher specimen was deposited at the Herbarium of Institute of Biology, University of Campinas (UEC number 181.451). As* P. umbellatum* is a Brazilian native genetic material, the present study had been approved by the Genetic Patrimony Management Board (CGEN/MMA), through Access and Shipment Component of Genetic Heritage for scientific research purpose (number 010646/2012-4).

### 2.2. Dichloromethane Crude Extract Production

Milled fresh leaves (1 kg) were extracted by maceration with dichloromethane (Dinamica) (1 : 5 leaves : solvent, 3 × 90 min) at room temperature. After filtration, the filtrates were pooled, evaporated (40°C, BUCHI model RE 215), and lyophilized (Virtis, model 8L) until dryness, affording DCE (2% yield).

### 2.3. Isolation of 4-Nerolidylcatechol

DCE was previously cleaned up for pigments and other lipophilic compounds (1 g) through liquid partition with hexane : acetonitrile (1 : 1) (3 × 100 mL). The acetonitrile phase (680 mg) was then applied on a solid-phase extraction (SPE) cartridge C18-E (55 *μ*M, 70 A, and 5 g/20 mL) Phenomenex previously conditioned with 10 mL methanol and 10 mL water, at 5 mL/min flow rate. SPE cartridge was eluted with 2 × 10 mL water : methanol (95 : 5, 50 : 50, 85 : 15, and 0 : 100, named as FA, FB, FC, and FD, resp.), at 3.5 mL/min flow rate. Fraction FC (190 mg) was analysed by RMN^1^H and ^13^C.

### 2.4. Chromatographic Analysis

HPLC analysis followed a previously described protocol [17]. It was performed with a Shimadzu series HPLC system equipped with online degasser (DUG-2A), quaternary pump (LC-10AT), autosampler (SIL 20A HT), column heater (CTO 10AS Vp), and photodiode array detector (SPD-M10Vp), using a C18 column (4.6 mm × 250 mm, 5 *μ*m particle size, Gemini, Phenomenex, Macclesfield, UK). Instrument control and data analysis was carried out using software* Class* VP 6.13 edition. The isocratic mobile phase was methanol-acetonitrile-water (62 : 20 : 18). Flow was set at 1.0 mL/min, injection volume was 20 *μ*L, and ultraviolet detection was at 282 nm.

### 2.5. Quantification of 4-Nerolidylcatechol

4-NC was quantified in the DCE by analytical curve. Stock solutions (2396 *μ*g/mL) were prepared in methanol and successively diluted in the range of 48 to 957 *μ*g/mL, two replicates each, in methanol. All samples were analyzed by HPLC as described in Chromatographic Analysis. A graphic correlating area under the curve (AUC) with the respective concentration was plotted and analyzed by linear regression using MS Excel software (Supplementary Figures  S1 and S2 in Supplementary Material available online at http://dx.doi.org/10.1155/2015/948737). After quantification, DCE was defined as standardized dichloromethane extract (SDE).

### 2.6. *In Vitro* Antiproliferative Assay

#### 2.6.1. Cell Lines

Human tumor cell lines (UACC-62 (melanoma), U251 (glioma), MCF-7 (breast), NCI-H460 (lung, non-small cells), HT-29 (colon), PC-3 (prostate), 786-0 (kidney), NCI-ADR/RES (ovarian expressing multiple drugs resistance phenotype), and OVCAR-3 (ovary)) were kindly provided by the National Cancer Institute (Frederick, MA, USA). Nontumor cell line HaCat (human keratinocytes) was donated by Professor Dr. Ricardo Della Coletta, FOP/UNICAMP.

#### 2.6.2. Cell Culture

Stock cultures were grown in medium RPMI 1640 (GIBCO) supplemented with 5% fetal bovine serum (FBS, GIBCO) and 10 U/mL penicillin, 10 *μ*g/mL streptomycin at 37°C in 5% CO_2_.

#### 2.6.3. Antiproliferative Assay

Cells in 96-well plates (100 *μ*L cells/well) were exposed to SDE (0.25, 2.5, 25, and 250 *μ*g/mL in DMSO/RPMI) at 37°C, 5% of CO_2_ in air for 48 h. Doxorubicin (DOXO) was used as standard (0.025, 0.25, 2.5, and 25 *μ*g/mL). Final DMSO concentration did not affect cell viability (0.25%). Before (T0 plate) and after (T1 plates) sample addition, cells were fixed with 50% trichloroacetic acid and cell growth was determined by spectrophotometric quantification (540 nm) of cellular protein content using sulforhodamine B (SRB) assay [18]. The TGI (concentration that produces total growth inhibition) was determined through nonlinear regression analysis using the concentration-response curve for each cell line in the software ORIGIN 8.0 (OriginLab Corporation) [19].

### 2.7. *In Vivo* Assays

#### 2.7.1. Animals

Experiments were conducted with Balb/C and Swiss female mice (20–30 g, 90 days old) from the Multidisciplinary Centre for Biological Investigation on Laboratory Animals Sciences (CEMIB, UNICAMP). Animals were maintained at the Animal Facilities of Pharmacology and Toxicology Division, CPQBA, UNICAMP (Paulínia, SP, Brazil), in a room with controlled temperature 25 ± 2°C for 12 h light/dark cycle, with free access to food and water. Animal care and research protocols were in accordance with the principles and guidelines adopted by the Brazilian College of Animal Experimentation (COBEA). Protocols were approved by the Ethical Committee for Animal Research (CEUA), Institute of Biology, UNICAMP (numbers 2868-1, 3182-1, 3052-1, and 3183-1). Euthanasia was performed by deeping anaesthesia followed by cervical dislocation.

#### 2.7.2. Drugs

The used drugs were Indocid (indomethacin 50 mg; Merck Sharp & Dohme), carrageenan (Sigma-Aldrich, EUA), and dexamethasone (Sigma-Aldrich, EUA). SDE was emulsified in Tween 80 (Sigma) 0.3% and dissolved in PBS, pH 7.0. Vehicle was PBS, pH 7.0 + Tween 80 (Sigma) 0.3%.

#### 2.7.3. Acute Toxicity

Swiss mice (*n* = 5) were fasted for 12 h and then treated orally with SDE 1000 and 2000 mg/kg. Groups were observed during 4 hours and then daily for 15 days, for general toxicity signals evaluation: body weight loss, locomotion, behaviour (agitation, lethargy), respiration, salivation, tearing eyes, cyanosis, and mortality [20].

#### 2.7.4. Subchronic Toxicity

Balb/C mice (*n* = 6) were treated orally with vehicle and SDE (100, 200, and 400 mg/kg), daily, for 21 days. Mice were weighed (every three days) and daily observed for possible signals of toxicity [20]. At the 21st day, whole blood was collected from the retroorbital plexus of each animal for complete blood count analyses (Sysmex model Poch-100iV) evaluating total leukocytes (WBC), erythrocytes (RBC), and platelets (Pt) count. Animals were euthanized and liver, spleen, and kidneys were macroscopically evaluated and weighed.

#### 2.7.5. Ehrlich Solid Tumor Assay

*Cells Maintenance and Preparation*. Ehrlich tumor cells were maintained in the ascitic form in Swiss mice by weekly transplantation of 5 × 10^5^ cells/animal in PBS (pH 7.0) [21]. For the experiments, cells were prepared at the density of 1 × 10^6^ cells/50 *μ*L/animal in PBS [22] after count in Neubauer chamber with trypan blue, to exclude nonviable cells and debris.

*Induction and Treatments*. Ehrlich cells suspension (1 × 10^6^ cells/50 *μ*L/animal) was inoculated subcutaneously in the flank of Balb/C mice (*n* = 8). On the 5th day, animals with palpable tumors were randomly divided into negative control (vehicle) and experimental (SDE: 100, 200, and 400 mg/kg) groups that were treated every day, orally, for 12 days. On the 17th day, animals were euthanized and tumors were removed and weighted. The relative tumor weight was calculated as tumor weight divided by corporal weight. The growth inhibition ratio was calculated according to the formula [(*A* − *B*)/*A*] × 100, where *A* is mean relative tumor weight of negative control group and *B* is mean of relative tumor weight from treated group [22].

#### 2.7.6. Carrageenan-Induced Paw Edema

Experiments were designed according to Posadas et al. [23] with modifications. Right hind paw basal volume of Balb/C mice (*n* = 8) was measured using a caliper (Mitutoyo) according to the ellipse oblate formula: *V* = (4/3)*πa* ^2^ *b*, where *a* is the paw laterolateral width and *b* is the dorsal-ventral width. Then animals were randomly divided into negative control (vehicle), positive control (indomethacin, 10 mg/kg), and experimental (SDE; 100, 200, and 400 mg/kg) groups, being orally treated one hour before inflammation induction by carrageenan solution inoculation (2.5 mg/mL, 40 *μ*L/animal) into the right hind footpad. The right footpad volume was evaluated 1.5, 3.0, 4.5, 6.0, 24, 48, and 72 h after carrageenan inoculation. Results were expressed as paw edema variations (mL, difference between measured and basal paw volumes) versus time.

#### 2.7.7. Carrageenan-Induced Peritonitis

Balb/C mice (*n* = 8) were randomly divided into negative control (vehicle), positive control (dexamethasone, 2.5 mg/kg), and experimental (SDE, 200 mg/kg) groups that were orally treated one hour before peritonitis induction by carrageenan solution inoculation (500 *μ*g/250 *μ*L/animal) into peritoneal cavity. Four hours later, mice were euthanized and the peritoneal cavity was washed with 5 mL of PBS containing heparin 5 IU/mL. Total leukocyte was analysed in peritoneal fluid using a haematology analyser (Sysmex model Poch-100iV).

### 2.8. Statistical Analyses

The results were presented as mean ± SEM. The statistical significance of difference between groups was assessed by one-way ANOVA, followed by Newman-Keuls post hoc test using GraphPad Prism 5.0 software. Values of *P* ≤ 0.05 were considered significant.

## 3. Results and Discussion

### 3.1. Quantification of 4-NC

4-Nerolidylcatechol (94% of analytical purity) was identified by experimental data comparison with those reported by Baldoqui et al. [11]. In our study, HPLC-DAD quantitative analysis (correlation coefficient *R* ^2^ = 0.9995 ± 0.0005; detection limit (LOD) = 11.6 *μ*g/mL; quantification limit (LOQ) = 35.1 *μ*g/mL) showed that* P. umbellatum* SDE presented 23.9% of 4-NC, considering the initial fresh leaves amount. 4-NC was selected as a chemical marker for the extract standardization since this compound is readily isolated and easily quantified both by HPLC-UV-DAD and by GC/MS. Moreover, due to the well-known potent antioxidant activity of 4-NC, this substance may be involved in the possible anti-inflammatory activity of SDE.

### 3.2. *In Vitro* Antiproliferative Assay

SDE showed a potent antiproliferative activity, as it promoted total growth inhibition of almost all tumor cell lines (TGI values between 6.8 and 14.9 *μ*g/mL), excepting HT-29 cell line (colon, TGI = 207.3 *μ*g/mL) (Table 1). Moreover, TGI value (144.6 *μ*g/mL) for HaCaT cells (nontumor cell line) was higher than those observed for most of the tumor cell lines, thus suggesting selectivity for tumor cells. These promising* in vitro* antiproliferative results were in accordance with our previous work [8] and prompted the study in* in vivo* models.

Considering SDE chemical composition, the observed antiproliferative effect could be partially attributed to the presence of 4-NC and sterols *β*-sitosterol, stigmasterol, and campesterol, as these compounds had been identified in* P. umbellatum* dichloromethane extracts by Sacoman et al. and Lopes et al. [4, 8].

*β*-Sitosterol induces apoptosis and G2/M arrest in MDA-MB-231 (breast), PC-3 (prostate), and HCT (colon) human tumor cell lines [24]. A proapoptotic activity of *β*-sitosterol was also reported by Moon et al. [25] in murine fibrosarcoma cells and human leukaemia. Moreover, 4-NC also induces changes in the cell cycle profile of SK-Mel-147 (melanoma), promoting a G1 arrest [26]. It is interesting to notice that Sacoman et al. [8] observed a higher* in vitro* antiproliferative effect for the steroids fraction compared to the 4-NC fraction. Similarly, Lopes et al. [4] observed that the dichloromethane extract was more potent than the 4-NC and sterol fractions in an* in vitro* antioxidant activity model, hypothesizing a synergic activity of these compounds.

### 3.3. *In Vivo* Assays

In view of confirming the* in vitro P. umbellatum* antiproliferative effect, the SDE was evaluated* in vivo* in the Ehrlich solid tumor model in mice. Previous studies with* P. umbellatum* DCE described its* in vivo* activity in the Ehrlich ascitic tumor model after intraperitoneal treatment [8]. This model allows evaluation of life span; however, it presents a limitation: when treatments are conducted by intraperitoneal route, samples are applied at the same place of Ehrlich tumor cells growth. This way, it is difficult to elucidate parameters related to sample absorption and distribution. Herein, we described the systemic effects of* P. umbellatum* SDE, as treatments were performed by oral route and tumor cells were implanted subcutaneously in the flank of the animals.

### 3.4. Acute and Subchronic Toxicity

Before the* in vivo* anticancer and anti-inflammatory experiments, an acute toxicity evaluation was conducted in order to determine the maximum tolerated dose (MTD) that could be used in the long-term studies without adverse effects. No evidence of toxicity was observed up to 4 hours after administration of SDE 1000 mg/kg by oral route, as well as during the following 14 days, when the animals were kept under observation. However, animals treated with 2000 mg/kg died after 4 hours. Therefore, MTD was determined as 1000 mg/kg for single treatment and to determine doses for repetitive treatments we considered the higher dose as 40% of MTD, as described by Mi et al. [27], together with two lower doses. This way,* in vivo* experiments were carried out with 100, 200, and 400 mg/kg of SDE, by oral route.

In our previous study, a lethal dose 50% (LD_50_) of 533.71 mg/kg was determined for single treatment with* P. umbellatum* DCE by intraperitoneal route [8]. Herein, oral LD_50_ of SDE could be considered in the range of 1000 to 2000 mg/kg. Such loss of toxicity after changing the treatment route (intraperitoneal to oral route) may suggest that the substances responsible for adverse effects in SDE could show low bioavailability and/or be quickly metabolized when administrated by oral route.

Moreover, when mice were treated every day, during 21 days, with* P. umbellatum* SDE 100, 200, and 400 mg/kg, no toxic signals and no haematological alterations were observed (Table 2). As most chemotherapeutic agents induce collateral effects, the observed results for* P. umbellatum* SDE were encouraging.

### 3.5. *In Vivo* Ehrlich Solid Tumor Assay

Solid tumors are structures resembling organs in their complexity and heterogeneity. Inside these tumors there are differences in pH, oxygen pressure, and nutrient flux, which often contribute to tumor resistance to chemotherapy due to irregular drugs distribution inside the tumor matrix. Therefore, the development of experimental models to complement* in vitro* drug screening is necessary due to the limitations inherent to cell cultures to predict the behaviour of solid tumors to chemotherapy [28, 29].

The* in vivo* anticancer activity of* P. umbellatum* SDE was evaluated in the Ehrlich solid tumor model in mice. Ehrlich tumor is an aggressive and fast growing murine breast adenocarcinoma, which is able to develop both in the ascitic and in the solid form, depending on whether it is inoculated (intraperitoneally or subcutaneously, resp.) [30]. Ehrlich tumor cells generate a local inflammatory response characterized by increased vascular permeability, which accounts for edema formation, cell migration, and recruitment of the immune response [31].

**Figure 1:**
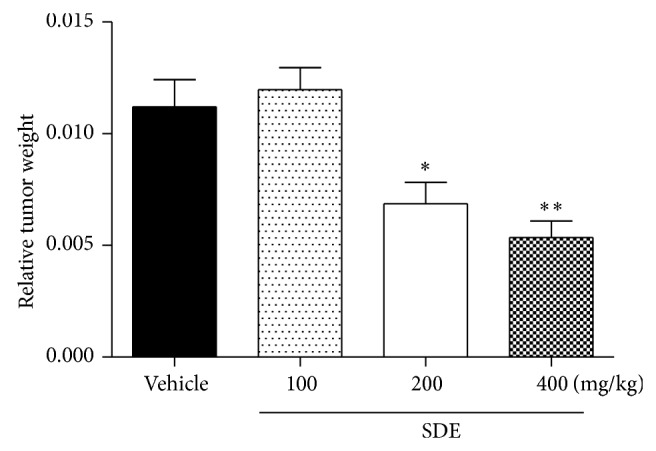
Relative tumor weight of Ehrlich solid tumor after treatment with vehicle and* P. umbellatum* SDE. Relative tumor weight was expressed as tumor weight divided by body weight; groups (*n* = 8) were treated daily (during 12 days) by oral route with vehicle (PBS, pH 7.0 + Tween 80 0.3%) and SDE 100, 200, and 400 mg/kg; ANOVA, Newman-Keuls Multiple Comparison Test, ^*^ *P* < 0.05, ^**^ *P* < 0.01 significantly different from negative control group (vehicle).

In the end of experiment, the relative tumor weight for the negative control group was 0.011 ± 0.0012, which was decreased in 38.7 and 52.2% (*P* < 0.05) after daily treatments with 200 and 400 mg/kg of* P. umbellatum* SDE, without signals of toxicity, while treatment with 100 mg/kg was not effective (Figure 1). These results are in accordance with those previously described by our group [8], with the advantage of loss of toxicity by changing route and treatment frequency. As discussed for* P. umbellatum* SDE* in vitro* antiproliferative activity, 4-NC and sterols present in SDE could be partly responsible for SDE* in vivo* antitumor activity.

Solid tumors are among the leading death causes in western countries, with growing incidence every year. Although the prognosis of these patients has been evolved because of early diagnosis and new antitumor therapies, there is still a need for new treatments [32]. Therefore, inhibition of tumor development by* P. umbellatum* SDE associated with low toxicity is an exciting result.

Certain types of cancers induce an inflammatory microenvironment formation, which contributes to tumor development [33]. As previously mentioned, Hanahan and Weinberg [13] included inflammation as a facilitator process, as it provides bioactive molecules such as growth, survival and angiogenic factors, and enzymes that modify the extracellular matrix, among others. In some cases, inflammation is already evident in early stages of carcinogenesis, by promoting tumor development since the action of inflammatory cells can lead to mutagenic agents' release [34].

In view of the relationship between cancer and inflammation, we evaluated* P. umbellatum* SDE anti-inflammatory potential in experimental inflammation models in mice.

### 3.6. *In Vivo* Anti-Inflammatory Assays

The administration of carrageenan 2.5% into the mouse hind footpad induces a biphasic inflammatory edema [35]. Immediately after carrageenan injection, there is a cascade of mediators' release, as histamine, serotonin, bradykinin, and phospholipase A_2_ (PLA_2_). These mediators promote an increase in vascular permeability and signal for arachidonate metabolites (prostaglandins, leukotrienes) and nitric oxide release, until the 6th hour. The second phase of inflammation starts after 24 hours, coinciding with a decrease in edema, but an increase in leukocytes migration, which amplify the inflammatory response and promote a second edema peak within 72 h [23].

**Figure 2:**
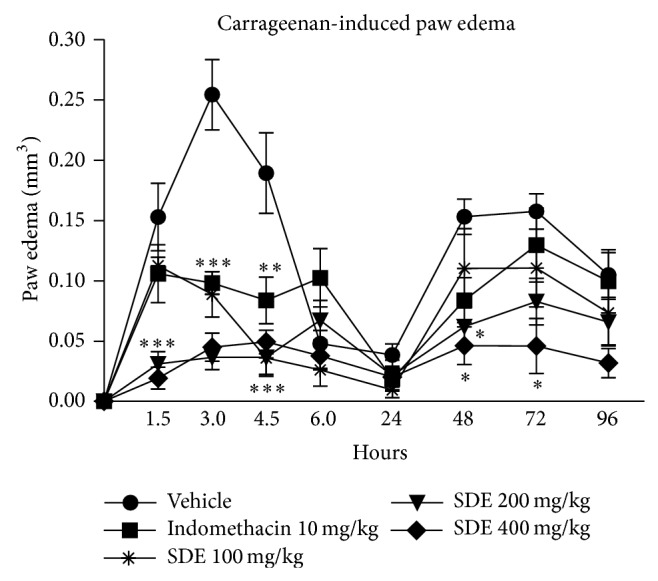
Anti-inflammatory effect of* P. umbellatum* SDE versus time after inflammatory stimulus on carrageenan-induced paw edema. Paw edema was measured with a caliper; results were expressed as paw edema in mm^3^ (mean ± SEM); treatments:* P. umbellatum* SDE (100, 200, and 400 mg/kg), vehicle (PBS, pH 7.0 + Tween 80 0.3%), or indomethacin (10 mg/kg) one hour before intraplantar carrageenan 2.5% injection. *n* = 8 animals/group. ANOVA, Newman-Keuls Multiple Comparison Test; ^*^ *P* < 0.05, ^**^ *P* < 0.01, and ^***^ *P* < 0.001 in comparison to negative control group (vehicle).

*P. umbellatum* SDE treatment significantly inhibited the first phase of inflammation, in an independent-dose way, as well as indomethacin 10 mg/kg (Figure 2 and Table 3). SDE was able to inhibit inflammation up to 4.5 hours, period coincident with prostaglandin release, which could suggest an action on prostaglandins production (Figure 2 and Table 3). In the second phase, all SDE doses inhibited inflammation at 48 hours while 400 mg/kg dose also inhibited the second inflammatory peak (72 h). This result suggests an effect on neutrophil mobilization, quite similar to the corticosteroids effects that efficiently inhibit the cellular phase of inflammation.

Previous studies performed with* P. umbellatum* ethanolic extract demonstrated its anti-inflammatory activity, with inhibition of the first phase of inflammation [5]. Another study showed the anti-inflammatory activity of a *β*-sitosterol rich fraction obtained from* Sideris foetens*, which was able to inhibit paw edema increase from 3 to 7 hours after inflammatory stimulus [36]. According to these authors, *β*-sitosterols could be responsible for the inhibition of arachidonate metabolites generation and neutrophil migration phase. This way, the anti-inflammatory effect herein described for* P. umbellatum* SDE, at higher dose, could be partly explained by the presence of sitosterols derivatives. Additionally, Núñez et al. [37] observed that* P. umbellatum* ethanolic crude extract and 4-NC inhibited the PLA_2_ enzymatic activity, which could also explain the SDE inhibitory effect on the first phase of inflammation (arachidonate metabolites generation).

Cytotoxic agents may inhibit the cellular phase of inflammation as demonstrated by Vendramini-Costa et al. [38]. These authors showed that doxorubicin inhibited the second phase of carrageenan-induced inflammation (after 24 hours of inflammation induction), which can be due to its cytotoxic effect on leukocytes, thus inhibiting their migration. As* P. umbellatum* SDE inhibited the second phase of carrageenan-induced inflammation (Figure 2) and tumor cell proliferation (Table 1 and Figure 1), we performed the carrageenan-induced peritonitis model to evaluate SDE activity on leukocyte migration.

Carrageenan when inoculated in the peritoneum exerts a chemotactic effect on inflammatory cells mediated by a synergistic action between prostaglandins, leukotrienes, and other chemotactic agents, producing a sustained increase in postcapillary venule permeability, which leads to cellular infiltration [39].

**Figure 3:**
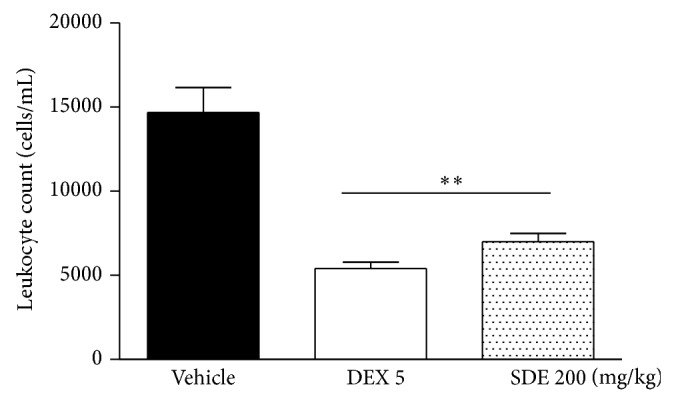
Effect of* P. umbellatum* SDE on carrageenan-induced peritonitis, expressed as leukocyte count (cells/mL). Results were expressed as mean ± SEM (*n* = 8 animals/group); treatment:* P. umbellatum* SDE (200 mg/kg), vehicle (PBS, pH 7.0 + Tween 80 0.3%), or dexamethasone (5 mg/kg), one hour before intraperitoneal carrageenan (500 *μ*g/250 *μ*L) injection. Peritoneal fluid was collected 4 hours after carrageenan stimulus. ANOVA, Newman-Keuls Multiple Comparison Test, ^**^ *P* < 0.01 in comparison to negative control group (vehicle).

In the carrageenan-induced peritonitis model, leukocytes migration in the negative control group was 14160 ± 1705 cells/mL and cell migration was inhibited both by dexamethasone (60.5%, 5 mg/kg) and by* P. umbellatum* SDE (52.0%, 200 mg/kg), *P* < 0.01 (Figure 3). These results corroborated that SDE could inhibit PLA_2_ activity in a similar way as dexamethasone. PLA_2_ is involved in arachidonic acid release from membrane phospholipids, which can be metabolized by cyclooxygenase (COX), lipoxygenase (LOX), and cytochrome P450 enzymes [34].

Based on the results presented here, we conclude that* P. umbellatum* SDE has promising antitumor and anti-inflammatory activities, without side effects even in high doses. In line with* P. umbellatum* SDE profile on paw edema and peritonitis model and previous reports on PLA_2_ inhibition, we hypothesize that SDE interferes on arachidonate metabolites generation, by inhibiting PLA_2_ or COX-2 activities. Further studies will be performed to clarify the biochemical pathways involved in these activities. These results highlight the importance of* Piper umbellatum* as a potential source of compounds against cancer and inflammation.

## Supplementary Material

4-NC was quantified by HPLC as described in "2.4. Chromatographic Analysis" (Figure S2) and "2.5. Quantification of 4-Nerolidylcatechol." in the DCE. The analytical curve plotted as described in "2.5. Quantification of 4-Nerolidylcatechol." and it was shown in Figure S1.

## Figures and Tables

**Table 1:** Concentration (*µ*g/mL) of *Piper umbellatum* SDE and doxorubicin (DOXO) required for total growth inhibition of cell lines (TGI values^a^).

Cell lines	Total growth inhibition (*µ*g/mL)	
	DOXO	SDE
UACC-62	0.9	6.8
U251	1.6	8.2
MCF-7	0.2	9.3
NCI-ADR/RES	1.9	14.9
786-0	1.1	9.1
NCI-H460	1.9	11.5
PC-3	1.9	8.2
OVCAR-3	1.2	8.3
HT-29	6.2	207.3
HaCat	29.1	144.6

^a^TGI values were determined by nonlinear regression analysis using ORIGIN 8.0 (OriginLab Corporation). Experiments were conducted in triplicate and results are representative of three different experiments.

**Table 2:** Cell blood count and organs weight (mean ± SEM) from animals treated (oral route) with vehicle and *P. umbellatum* SDE (100, 200, and 400 mg/kg) during 21 days.

Organs	Vehicle	100	200	400
Liver (g)	0.048 ± 0.001	0.045 ± 0.0010	0.046 ± 0.0029	0.051 ± 0.0009
Kidneys (g)	0.012 ± 0.0001	0.012 ± 0.0002	0.013 ± 0.0002	0.012 ± 0.0003
Spleen (g)	0.004 ± 0.0002	0.005 ± 0.0002	0.005 ± 0.0007	0.004 ± 0.0001

*Cell blood count *				
WBC (10^6^/*μ*L)	4.4 ± 0.3	3.3 ± 0.3	3.7 ± 0.7	4.7 ± 0.6
Haemoglobin (g/dL)	14.2 ± 0.3	14.2 ± 0.2	13.9 ± 0.5	13.9 ± 0.2
Platelet (10^3^/*μ*L)	1169 ± 44.5	1278 ± 28.9	1403 ± 76.3	1388 ± 61.9

Vehicle = PBS + Tween 80 0.3%, pH 7.0.

**Table 3:** Inhibitory effect of *P. umbellatum* SDE versus time after inflammatory stimulus on carrageenan-induced paw edema.

Treatments (mg/kg)	Time (hours) and % of inhibition							
	1.5	3	4.5	6	24	48	72	96
Vehicle	0.15 ± 0.03	0.25 ± 0.03	0.19 ± 0.03	0.05 ± 0.01	0.04 ± 0.001	0.15 ± 0.01	0.16 ± 0.01	0.10 ± 0.02

Indomethacin 10	0.11 ± 0.03 (28.7%)	0.10 ± 0.01 (60.6%)^***^	0.09 ± 0.02 (54.6%)^**^	0.10 ± 0.02 —	0.02 ± 0.001 (51.2%)	0.08 ± 0.02 (45.3%)	0.13 ± 0.03 (17.7%)	0.10 ± 0.03 (4.9%)

SDE 100	0.10 ± 0.02 (35.6%)	0.08 ± 0.02 (69.5%)^***^	0.05 ± 0.02 (74.5%)^***^	0.03 ± 0.01 (45.6%)	0.01 ± 0.01 (75.3%)	0.11 ± 0.03 (27.9%)	0.11 ± 0.03 (29.8%)	0.07 ± 0.03 (29.9%)

SDE 200	0.03 ± 0.01 (79.7%)^***^	0.04 ± 0.01 (83.1%)^***^	0.04 ± 0.01 (77.0%)^***^	0.07 ± 0.02 —	0.021 ± 0.01 (44.2%)	0.06 ± 0.02 (59.5%)^*^	0.08 ± 0.02 (47.5%)	0.065 ± 0.02 (37.3%)

SDE 400	0.02 ± 0.01 (82.4%)^***^	0.06 ± 0.01 (76.9%)^***^	0.058 ± 0.01 (67.8%)^***^	0.04 ± 0.01 (20.9%)	0.020 ± 0.01 (47.7%)	0.05 ± 0.02 (69.9%)^*^	0.05 ± 0.02 (70.9%)	0.03 ± 0.01 (69.8%)

Paw edema was measured with a caliper; results were expressed as paw edema in mm^3^ (mean ± SEM); treatments: *P. umbellatum* SDE (100, 200, and 400 mg/kg), vehicle (PBS, pH 7.0 + Tween 80 0.3%), or indomethacin (10 mg/kg) one hour before intraplantar carrageenan 2.5% injection, *n* = 8 animals/group. ANOVA, Newman-Keuls Multiple Comparison Test; ^*^ *P* < 0.05, ^**^ *P* < 0.01, and ^***^ *P* < 0.001 in comparison to negative control group (vehicle).

## Abbreviations

4-NC: 4-Nerolidylcatechol
SDE: Standardized dichloromethane extract
DCE: Dichloromethane crude extract
DMSO: Dimethyl sulfoxide
DOXO: Doxorubicin
TGI: Total growth inhibition
PBS: Phosphate buffered saline
ANOVA: One-way analysis of variance
SRB: Sulforhodamine B
MTD: Maximum tolerated dose
COX: Cyclooxygenase
LOX: Lipoxygenase
PLA_2_: Phospholipase A_2_.
