# Supplementary material for: Anticancer and Anti-Inflammatory Activities of a Standardized Dichloromethane Extract from Piper umbellatum L. Leaves
Source: Evid Based Complement Alternat Med. 2015 Feb 3;2015:948737. doi: 10.1155/2015/948737 (PMC4332971; doi:10.1155/2015/948737)
Supplement: Supplementary file 1 — 4-NC was quantified by HPLC as described in "2.4. Chromatographic Analysis" (Figure S2) and "2.5. Quantification of 4-Nerolidylcatechol." in the DCE. The analytical curve plotted as described in "2.5. Quantification of 4-Nerolidylcatechol." and it was shown in Figure S1. [file 948737.f1.pdf]

Supplementary information section

A.

concentration (µg/mL)	area
48	570203
96	1068015
193	2188404
240	2745412
288	3290756
479	5340609
719	8258301
957	11108126

B.

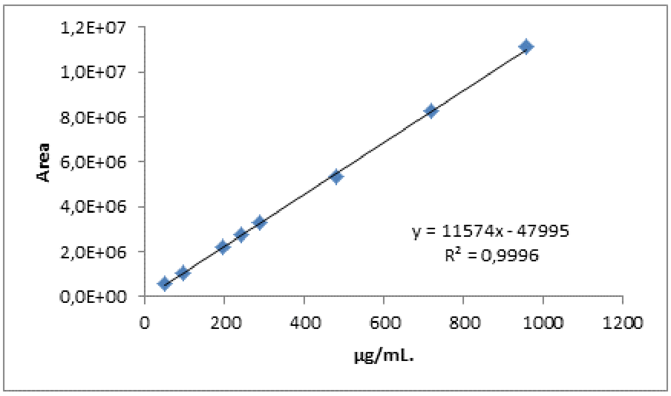

Figure S1. Analytical curve of 4-nerolidylcatechol. A. Stock solutions of DCE (2396 µg/mL) were prepared in methanol and successively diluted in the range of 48 to 957 µg /mL, two replicates each, in methanol. All samples were analyzed by HPLC. B. Correlation area under the curve (AUC) with the respective concentration of DCE.

Supplementary information section

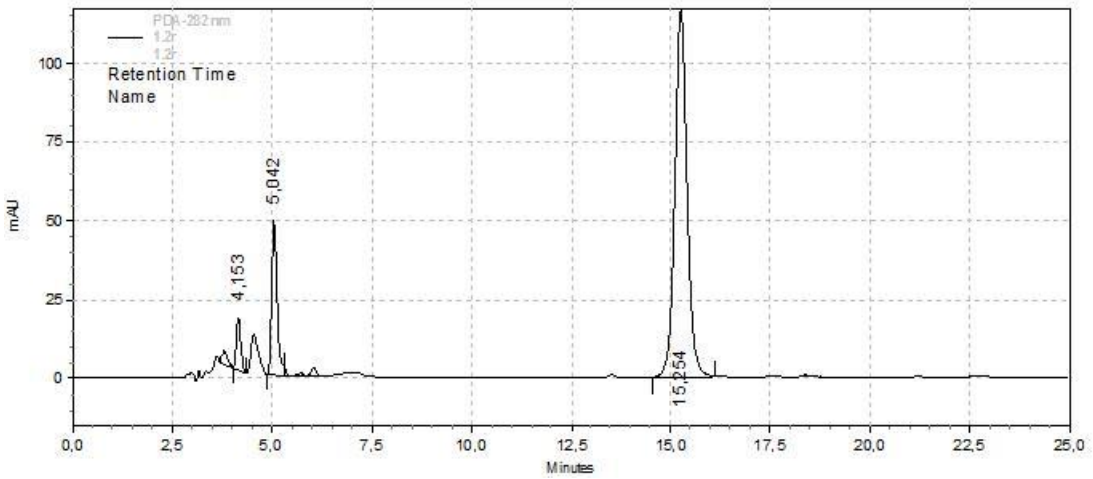

Figure S2: HPLC/DAD chromatogram of the dichloromethane crude extract from *P. umbellatum* leaves.
